# Supplementary material for: Cross-reactive Dengue virus-specific CD8+ T cells protect against Zika virus during pregnancy
Source: Nat Commun. 2018 Aug 2;9:3042. doi: 10.1038/s41467-018-05458-0 (PMC6072705; doi:10.1038/s41467-018-05458-0)
Supplement: Supplementary file 1 — Supplementary Information [file 41467_2018_5458_MOESM1_ESM.pdf]

Supplementary Figure Legends

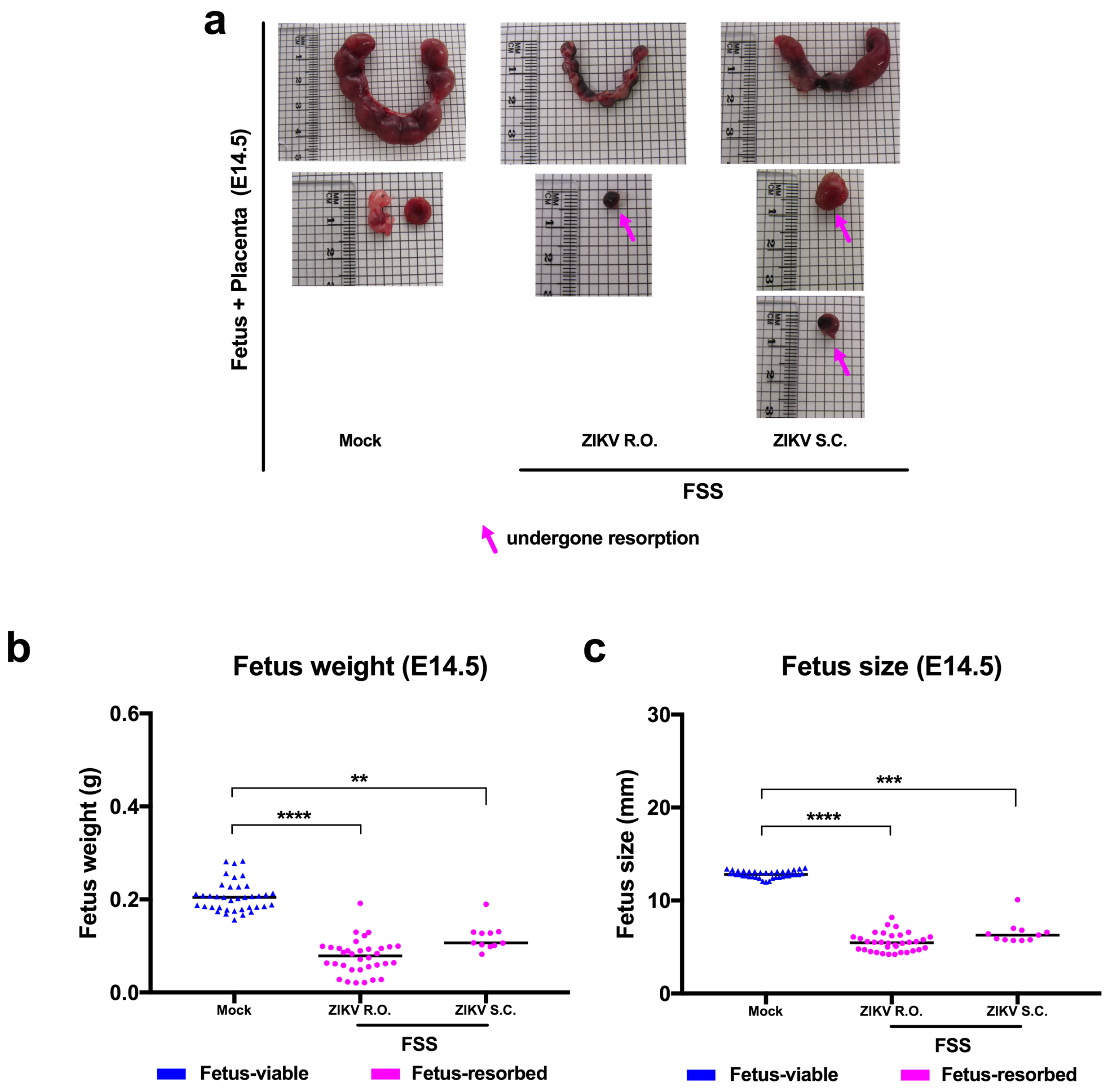

Supplementary Fig. 1, related to Figures 1-3. Fetal resorption in *Ifnar1*$^{-/-}$ dams with retro-orbital or intra-footpad infection with ZIKV.
Non-immune *Ifnar1*$^{-/-}$ dams were inoculated via retro-orbital (R.O.) or subcutaneous (via footpad) (S.C.) route at E7.5 with 10^4 FFU of ZIKV FSS13025 or 10% FBS-PBS as Mock. (**a**) Representative pictures from uterus, fetus, and placenta with decidua at E14.5 are shown. Magenta arrows indicate the presence of fetal resorption. (**b**) Fetal body weight and (**c**) size were measured at E14.5. n = 38 fetuses from 5 separate mothers (Non-immune-Mock), n = 34 fetuses from 4 separate mothers (Non-immune-ZIKV-R.O.), and n = 11 fetuses from 2 separate mothers (Non-immune-ZIKV-S.C.). Weight and size were determined individually on the residual placenta if fetal resorption was observed. Data were pooled from two independent experiments. All data were expressed as a median with interquartile range. ***p*<0.01, ****p*<0.001, *****p*<0.0001. Kruskal-Wallis test was used for **b and c.**

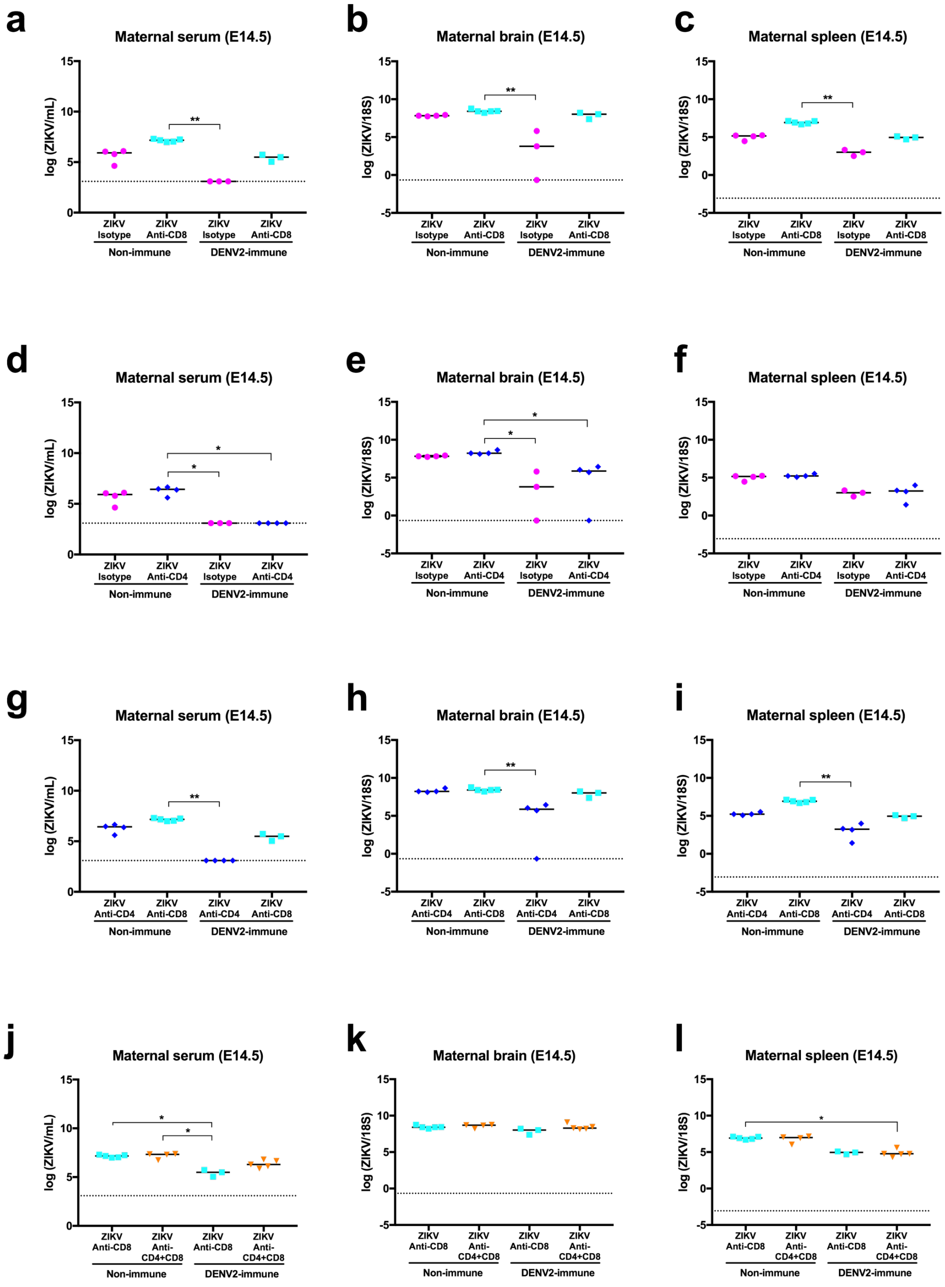

Supplementary Fig. 2, related to Figure 1. The effect of CD4⁺, CD8⁺, or combined CD4⁺ and CD8⁺ T cell depletion on ZIKV viral burden in non-immune and DENV2-immune *Ifnar1*$^{-/-}$ dams.

Non-immune and DENV2-immune *Ifnar1*$^{-/-}$ dams were depleted of $CD4^+$ and/or $CD8^+$ T cells and challenged with ZIKV at E7.5 as described in **Fig. 1**. ZIKV RNA levels in maternal serum, brain, and spleen harvested at E14.5 were quantitated by qRT-PCR. ZIKV RNA levels were compared among 4 groups including 3 different combinations of T cell depletion: (**a-c**) Isotype versus Anti-CD8. (**d-f**) Isotype versus Anti-CD4, (**g-i**) Anti-CD4 versus Anti-CD8, and (**j-l**) Anti-CD8 vs Anti-CD4+CD8. n = 4 separate mothers (Non-immune-ZIKV+isotype), n = 5 separate mothers (Non-immune-ZIKV+Anti-CD8), n = 3 separate mothers (DENV2-immune-ZIKV+isotype), n = 3 separate mothers (DENV2-immune-ZIKV+Anti-CD8), n = 4 separate mothers (Non-immune-ZIKV+Anti-CD4), n = 4 separate mothers (DENV2-immune-ZIKV+Anti-CD4), n = 4 separate mothers (Non-immune-ZIKV+Anti-CD8+CD4), and n = 5 separate mothers (DENV2-immune-ZIKV+Anti-CD8+CD4). Data were pooled from two independent experiments and were expressed as a median. *p<0.05, **p<0.01. Kruskal-Wallis test was used for **a-l**.

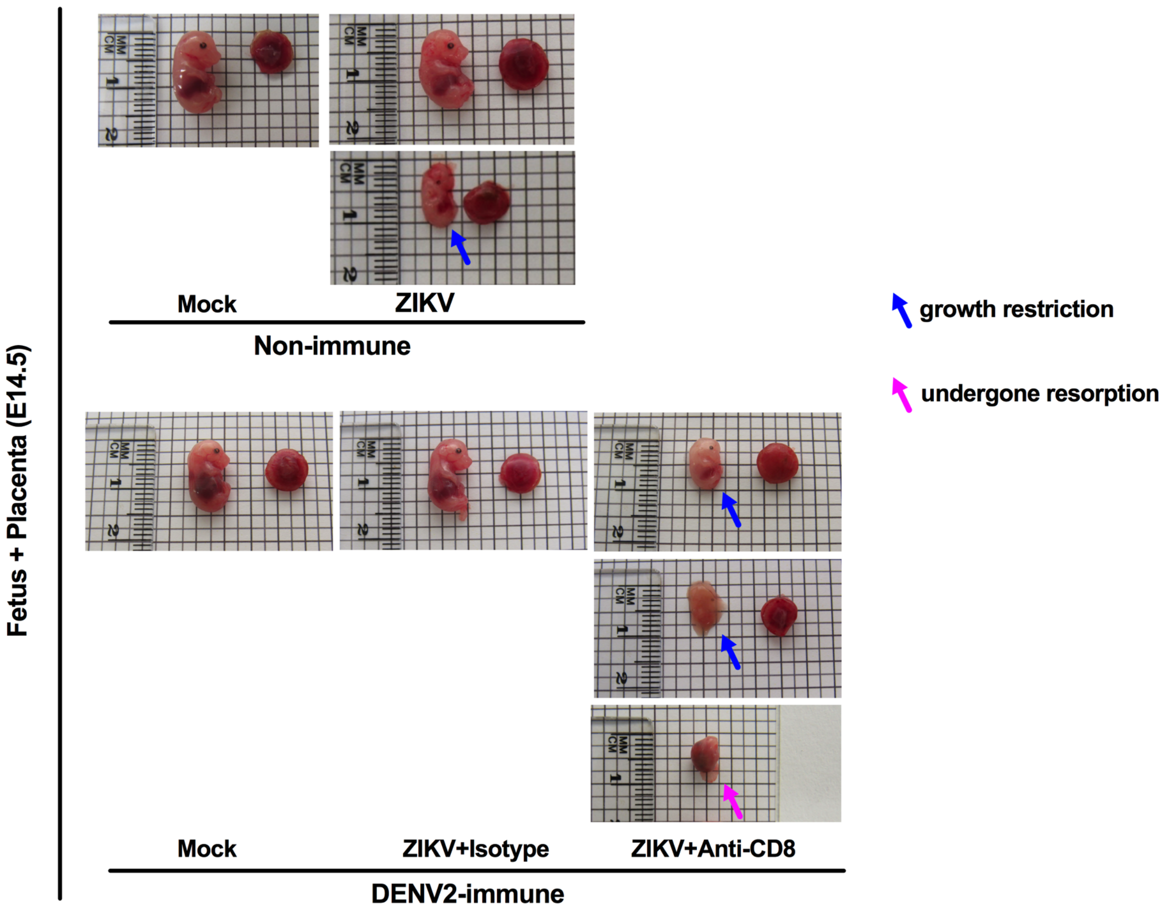

Supplementary Fig. 3, related to Figures 2, 4 and 5. Phenotype of fetuses at E14.5 from non-immune or DENV2-immune WT dams treated with Ifnar1-blocking Ab with or without CD8$^+$ T cell depletion.
Non-immune or DENV2-immune WT dams that were treated with Ifnar1-blocking Ab were challenged with ZIKV at E7.5 as described in **Fig. 2**. Tissues were harvested 7 days post-infection at E14.5. Representative images of fetuses and placentas from non-immune or DENV2-immune dams with or without anti-CD8 Ab administration are shown. n = 6 separate mothers (Non-immune-Mock), n = 5 separate mothers (Non-immune-ZIKV), n = 4 separate mothers (DENV2-immune-Mock), n = 5 separate mothers (DENV2-immune-ZIKV+isotype) and n = 4 separate mothers (DENV2-immune-ZIKV+Anti-CD8). Blue and magenta arrows indicate the presence of fetal growth restriction and resorption, respectively.

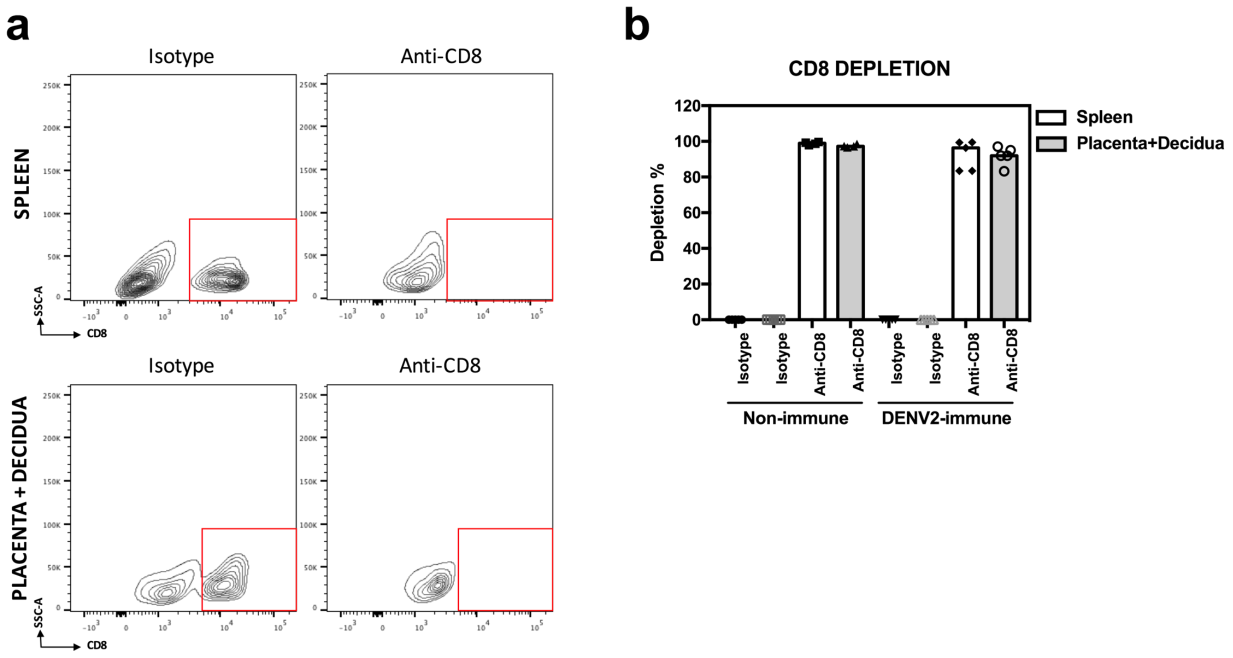

Supplementary Fig. 4, related to Figures 2-7. Efficiency of CD8$^+$ T cell depletion in WT dams administered anti-CD8 Ab.

Pregnant non-immune or DENV2-immune WT mice were challenged with ZIKV at E7.5 as described in **Fig 2.** (**a**) The gating strategy used to analyze the presence of CD8$^+$ T cells by flow cytometry in the spleen and decidua/placenta is illustrated. All plots represented were first gated on CD3$^+$ cells. (**b**) The percentages of CD8$^+$ T cell depletion in spleen and decidua/placenta are represented for isotype control or anti-CD8 Ab-treated dams in both non-immune and DENV-2 immune groups. Data are expressed as a median.

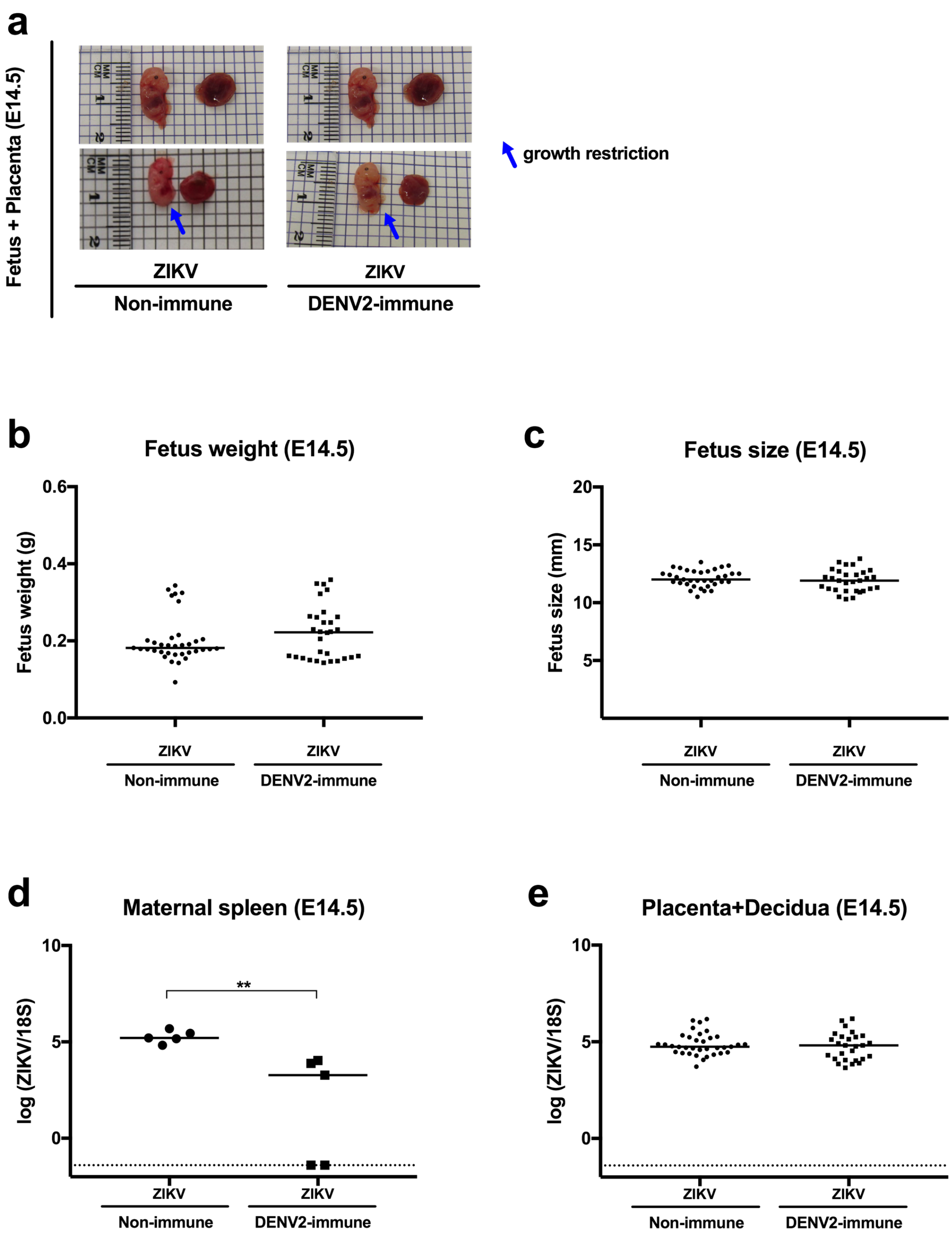

Supplementary Fig. 5, related to Figures 2 and 3. Phenotype of fetuses from DENV2-immune WT dams that were primed for 80 days prior to ZIKV challenge.

Non-immune and DENV2-immune WT dams that were primed for 80 days were treated with Ifnar1-blocking Ab and challenged with ZIKV at E7.5 as described in Fig. 2. Tissues were harvested 7 days after ZIKV infection at E14.5. (**a**) Representative images of fetuses and placentas are shown. n = 30 fetuses from 5 separate mothers (DENV2-immune-ZIKV) and n = 35 fetuses from 5 mothers (Non-immune-ZIKV). Blue arrows indicate fetal growth restriction. (**b**) Fetus weight and (**c**) size were recorded. (**d-e**) ZIKV RNA levels in the maternal spleen and placentas with decidua at E14.5 were measured by qRT-PCR. Data were pooled from two independent experiments. Data are expressed as a median. $**p<0.01$. Two-tailed Mann Whitney test was used.

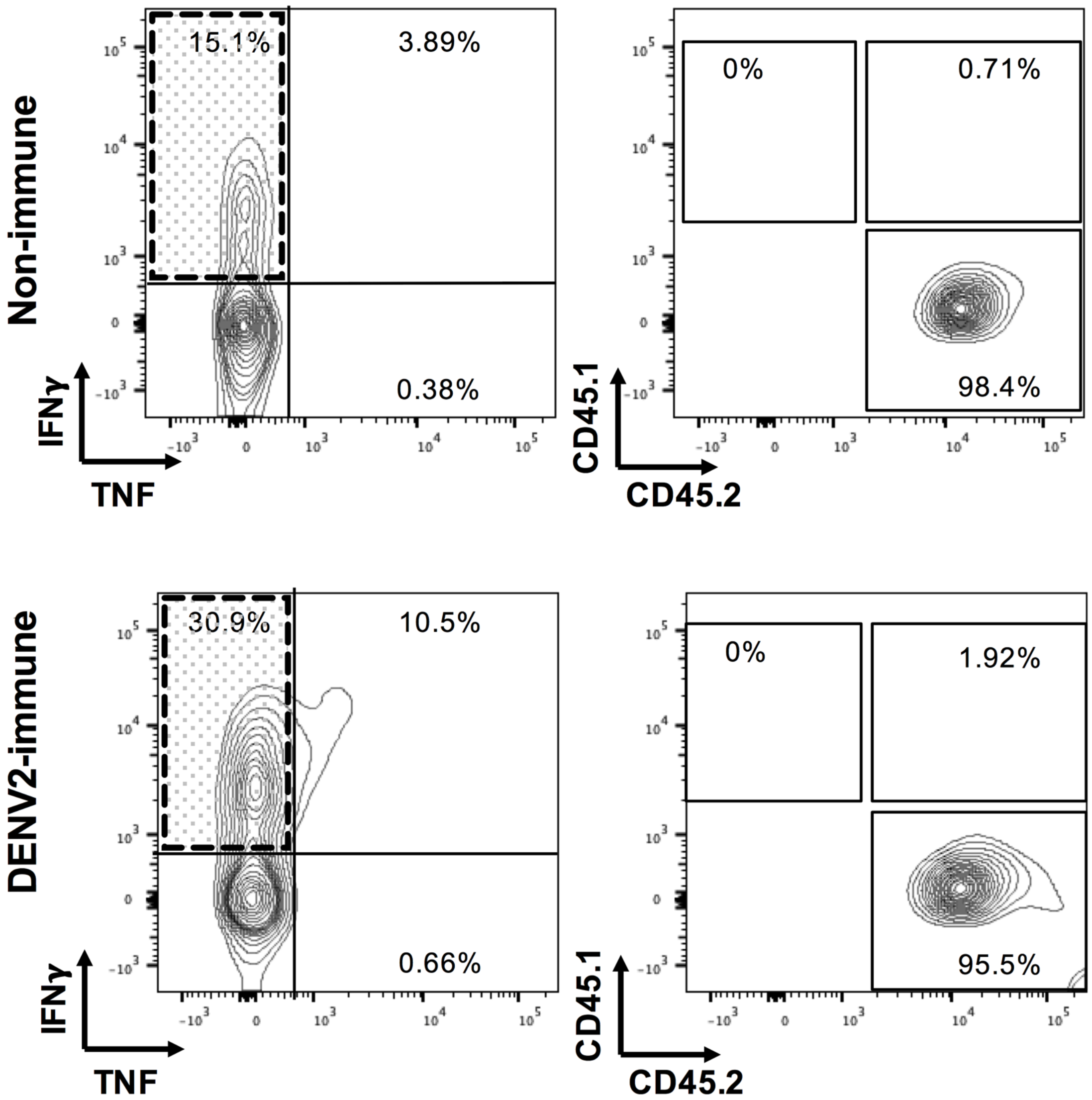

Supplementary Fig. 6, related to Figures 2, 3 and 7. Maternal origin of cross-reactive CD8$^+$ T cells in the decidua/placenta from WT mice treated with Ifnar1-blocking Ab.

CD45.1$^+$ WT sires were bred with non-immune or DENV2-immune CD45.2$^+$ WT dams, and cells from the placenta/decidua of ZIKV-infected dams were harvested at E14.5 as described in Fig. 2. Isolated cells were stimulated with a pool of 5 cross-reactive peptides for ICS and stained for cytokine production and CD45.1 and CD45.2 alleles. Representative plots of the gating strategy for IFNγ-, TNF-, and CD45.1/CD45.2-expressing antigen-specific CD8$^+$ T cells were illustrated for non-immune (n=4, top panel) and DENV2-immune groups (n=4, bottom panel). Two independent experiments were performed.
