# Supplementary material for: Cross-reactive Dengue virus-specific CD8+ T cells protect against Zika virus during pregnancy
Source: Nat Commun. 2018 Aug 2;9:3042. doi: 10.1038/s41467-018-05458-0 (PMC6072705; doi:10.1038/s41467-018-05458-0)
Supplement: Supplementary file 3 — Description of Additional Supplementary Files [file 41467_2018_5458_MOESM3_ESM.pdf]

Description of Additional Supplementary Files

File Name: Supplementary Data 1
Description: Number of animals used in each experiment (n).

File Name: Supplementary Data 2
Description: *P* values obtained after performing statistical tests.
